# Measuring and Comparing Municipal Policy Responses to COVID-19

**DOI:** 10.1017/S000842392000044X

**Published:** 2020-05-08

**Authors:** David A. Armstrong, Jack Lucas

**Affiliations:** 1 Department of Political Science, University of Western Ontario, London, Ontario, N6A 5C2; 2 Department of Political Science, University of Calgary, 2500 University Drive NW, Calgary, Alberta, T2N 1N4

## Abstract

Municipal governments are experts in social non-distancing. From swimming pools to libraries, streetcars to public parks, municipalities bring residents together and move them around—services vital to a vibrant community in ordinary times, but potentially disastrous in a pandemic. Municipal decisions to shutter these services and enforce social distancing are thus crucial for a successful COVID-19 response.

## Introduction

Municipal governments are experts in social non-distancing. From swimming pools to libraries, streetcars to public parks, municipalities bring residents together and move them around—services vital to a vibrant community in ordinary times, but potentially disastrous in a pandemic. Municipal decisions to shutter these services and enforce social distancing are thus crucial for a successful COVID-19 response.

Not only are these municipal policy decisions costly, but they also depend on a shared commitment to decisive and coordinated action. This shared commitment is by no means guaranteed; in the U.S., for example, municipal responses to the pandemic appear to vary quite substantially by region and local partisanship. Under normal circumstances, we would hardly be surprised to see variation in municipal policy decisions that involve substantial costs, serious collective action challenges, and potential ideological disagreement.

In this note, we use a survey of 551 councillors in 306 Canadian municipalities to measure the aggressiveness of municipal COVID-19 policy responses. We show that aggressiveness is strongly related to municipal population size and case totals and modestly related to province and local ideology. These findings reflect a widespread commitment among Canadian municipalities to aggressive policy action while also revealing important features of Canada's political geography.

## Municipal Policy Responses to COVID-19

The CMB COVID-19 survey, a survey of politicians in municipalities above 9,000 population, contained a number of factual questions about municipal responses to the COVID-19 pandemic.^1^ We asked if parks, libraries, city halls, recreation facilities, and public transit were open, partially closed, or fully closed; if council and committee meetings were cancelled, in camera, virtual, or proceeding as usual; and if the municipality had declared a state of emergency. These questions were modelled on data that had been collected for a smaller number of cities, and provided a useful overall picture of how municipalities are responding to the pandemic.^2^ We begin with an overview of variation in the individual measures and then describe our approach to using the structure in the data to build a summary measure of municipal policy aggressiveness.

### Descriptive Overview

In considering the individual policy instruments, we first want to highlight instances of near unanimity. Roughly 98 per cent of municipalities indicated that they had either cancelled or moved council meetings to in camera or virtual formats, and 100 per cent did the same for committee meetings. Only 1 per cent of municipalities have not fully closed recreation facilities and 5 per cent have not fully closed libraries.

On the remaining policies, we do see some variation across municipalities. For example, only 43 per cent of municipalities have fully closed city halls, and 65 per cent have fully closed their parks. Transit operations also remain at least partially open in most places. Only 13 per cent of municipalities have fully closed transit operations, and another 66 per cent have partially closed those operations. Finally, 41 per cent of municipalities have declared states of emergency.

**Figure 1.**
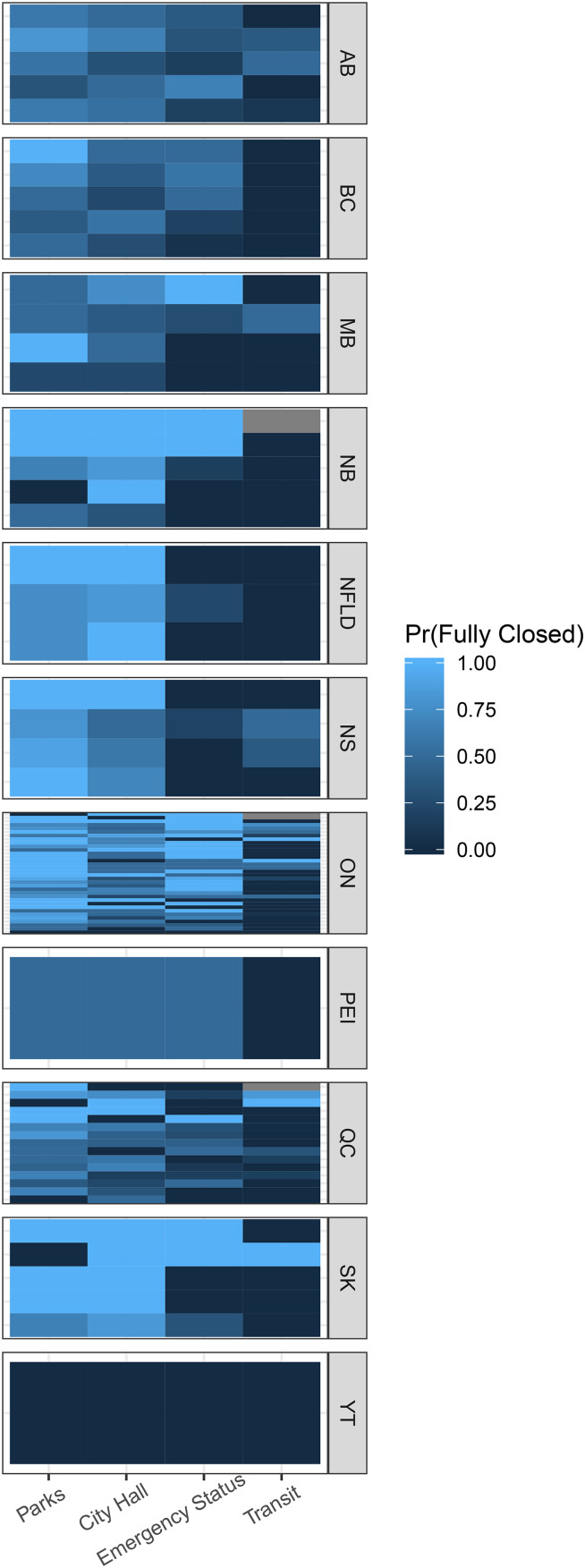
COVID-19 response by health unit.

To provide a sense of how these policy choices may vary, we plot policy adoption by health unit within provinces. Figure 1 shows the proportion of fully closed facilities (or emergency status declared) by health unit. The units are ordered within province by their mean across all of the indicators; on average, Ontario appears to have the most aggressive response (i.e., lightest blue colours). Provinces closer to the top have municipalities that have, on average, closed more services than those toward the bottom. There is also considerable within-health-unit variation in responses, which we explore in more detail below.

### Latent Variable Model of Policy Aggressiveness

The structure that is visible in the plot in Figure 1 suggests that we might think about each policy choice as existing on an underlying scale, which we might call the “aggressiveness” of the municipality's response. We can think of each policy as having a level of “difficulty,” where difficulty is understood to mean how much of the underlying trait (aggressiveness) a municipality needs to possess for us to predict that it would implement that policy. This allows us to array each policy on a single dimension, from less to more “difficult,” and to use this dimension to estimate a municipality's overall “aggressiveness.”

We use a Bayesian Item Response Theory model to identify and describe the underlying structure in the survey responses (Fox, 2010). Due to the lack of variation on the “parks” and “city hall” policies, we collapsed these into binary indicators of “fully closed” versus “partially closed/open” responses. The declaration of emergency is already a binary indicator. We maintain the three available categories on the transit indicator (open/partially closed/fully closed), as there is meaningful variation across all three.

We model the binary indicators using a logistic regression of the indicator on the latent variable. For the binary variables, our model is
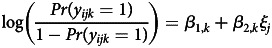


Here, *i* refers to the individual within each of the *j* municipalities for each of the *k* indicators. For the ordinal indicator (transit), we estimate
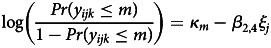
where *κ*_*m*_ is the threshold parameter for each category such that,
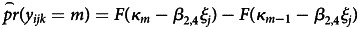


We make a number of identifying assumptions. We assume *κ*_0_ = −∞ and that *κ*_3_ = ∞ and that *κ*_*m*_ > *κ*_*m*−1_ to identify the ordinal logit model. We also use *β*_1,2_ = 1 and that *β*_1,1_ = *F*^−1^(*Pr*(city hall status = 1)) ≈ 0.166. We put a standard normal prior on the *ξ*_*j*_ (the latent variable estimates) and an uncorrelated bivariate normal prior with mean zero and variance of 10 on each of the *β*_*k*_ = {*β*_*k*,1_, *β*_*k*,2_}. After examining diagnostics, we are confident that the model converged.^3^

#### Summary of Distributions and Item Characteristic Curves

**Figure 2.**
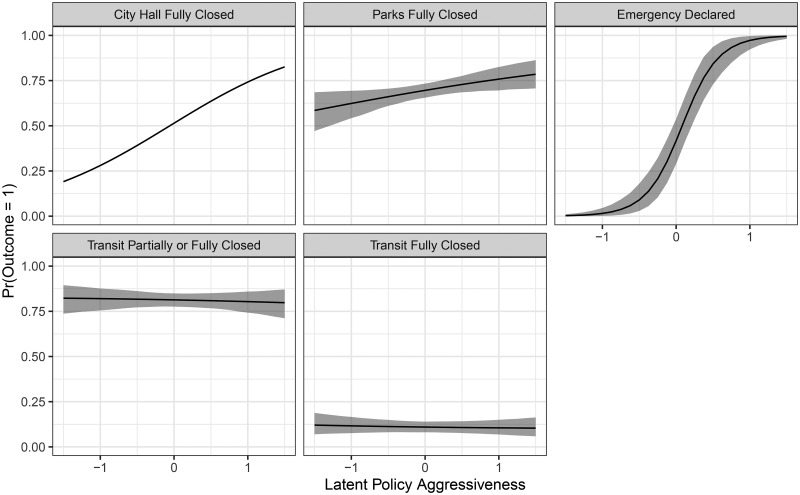
Item characteristic curves for the IRT model.

For each indicator in the model, we can plot item characteristic curves (ICC), which display the probability of each indicator outcome as a function of the latent variable. These curves, which we plot in Figure 2, show that the most reliable indicator is whether or not a state of emergency has been declared, though the status of city hall and parks also appears to help discriminate on the latent trait.^4^ Because the latent trait appears to correspond to increasing levels of response, we refer to the latent trait as policy aggressiveness.^5^ Municipalities with the least aggressive policies close city halls at a rate of about 25 per cent, while the most aggressive municipalities close their city halls at a rate of about 80 per cent. Parks are closed by the least aggressive municipalities just less than 60 per cent of the time, whereas the most aggressive municipalities close parks at a rate of about 80 per cent. The opening and closing of transit operations seems to be almost completely unrelated to aggressiveness of policy and is probably more directly related to local needs than the aggressiveness of the pandemic policy response. In terms of difficulty as defined above, we would say that at least partially closing transit is the least difficult, followed by closing parks, declaring a state of emergency, and fully closing transit operations, in that order.

**Figure 3.**
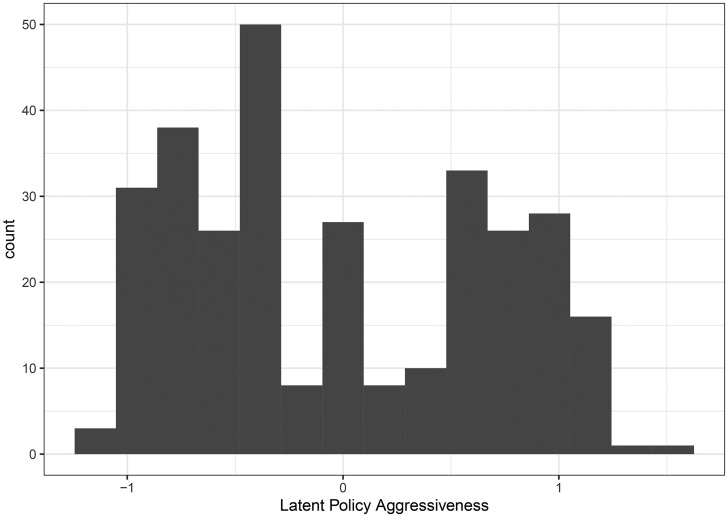
Distribution of policy aggressiveness.

Finally, the model allows us to visualize the overall distribution of aggressiveness in Canadian municipalities, which we do in Figure 3. The distribution appears to be bimodal, with one spike below the mean and one spike above—likely due to the importance of having declared an emergency.

## Patterns of Policy Aggressiveness

Our measure of municipal aggressiveness also allows us to explore relationships between municipal policy responses and potentially relevant features of the Canadian local context. Here we focus on four: province, population size, COVID-19 cases, and local ideology.

### Province

**Figure 4.**
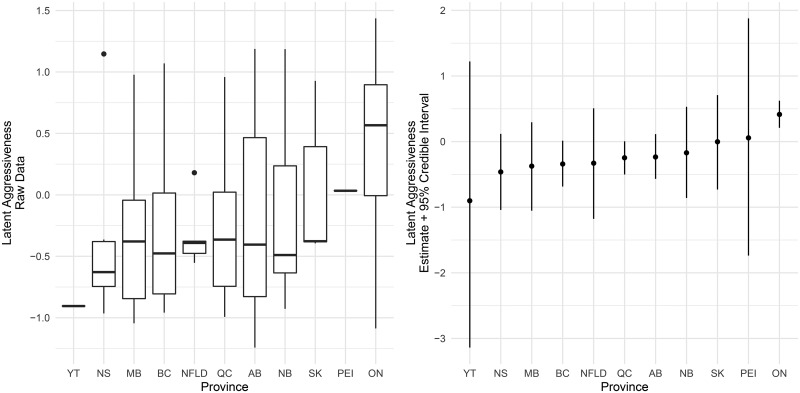
Municipal policy aggressiveness by province.

Canadian municipalities exist within provincial statutory-regulatory regimes and have been coordinating closely with provincial officials. We might therefore expect to see variation in municipal policy responses by province. The left panel in Figure 4 summarizes the distribution of latent aggressiveness values by province; for each province, the thick horizontal line marks the median aggressiveness value, and the box captures the interquartile range.^6^

One province, Ontario, clearly stands out from the others—a result, at least in part, of a much higher likelihood in Ontario of having declared a municipal state of emergency. The right panel of Figure 4 shows estimates of the average policy aggressiveness by province with 95 per cent credible intervals surrounding the estimates.^7^ Our follow-up research suggests that this difference is not a function of provincial incentives; one Ontario mayor told us the province explicitly assured him that funding was not contingent on a state of emergency.^8^ Instead, Ontario municipalities appear to have been more inclined than others to use states of emergency to communicate the seriousness of the pandemic to residents. They were also more common in Ontario because of regional coordination, where every municipality in a region simultaneously declared a state of emergency. A combination of this “communicative” role and Ontario's regionalized character appears to have prompted this more widespread use of states of emergency.^9^

### Population Size

**Figure 5.**
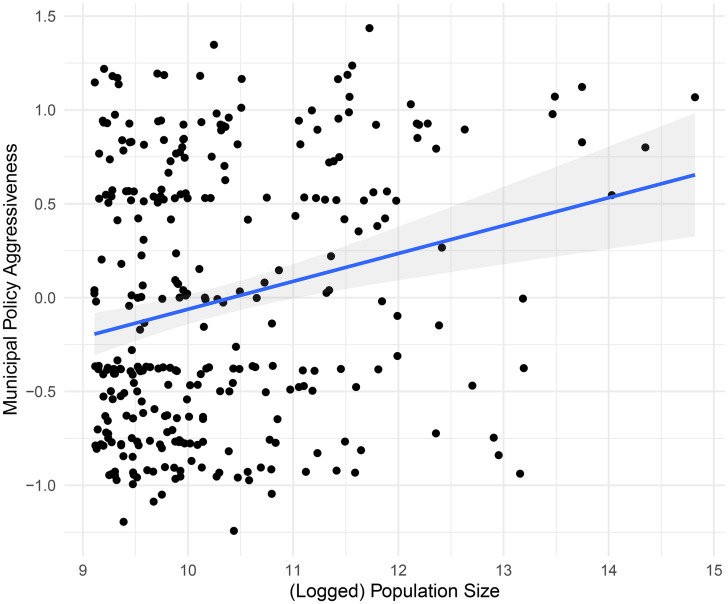
Municipal policy aggressiveness by population size.

Canada's biggest cities are especially hard-hit by the COVID-19 pandemic. In Figure 5, we see that larger cities are substantially more likely to have adopted more aggressive policy responses than smaller municipalities. Despite the enormous costs involved in these decisions, Canada's biggest cities have been very aggressive in their COVID-19 responses.^10^ This relationship is statistically reliable, as 99.6 per cent of the posterior distribution of the effect is greater than 0. The estimated effect (mean of the posterior distribution) is 0.149.^11^

### COVID-19 Cases

**Figure 6.**
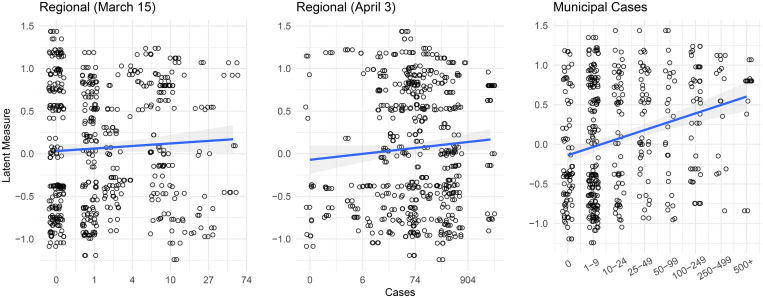
Municipal policy aggressiveness by COVID-19 cases.

We might also expect municipal policy responses to be closely related to the local seriousness of the pandemic. To explore this possibility, we matched each of our 306 municipalities to its corresponding health region—the most fine-grained geographic area for which COVID-19 case data are available. In Figure 6, we display the relationship between aggressiveness and regional health totals up through March 15 (when municipal responses began to ramp up) in the left-hand plot, and totals up through April 3 (the beginning of the CMB survey) in the middle plot. In both plots, we see little relationship between regional totals and municipal aggressiveness.^12^ Instead, what is most obvious is the variation in aggressiveness across the full span of case totals.

In the right-hand plot in Figure 6, we replace regional totals with *municipal* case totals supplied to us by municipal politicians themselves. While these reported totals cannot be verified against current official data, they do correspond closely to news reports of local case totals. On this more localized measure, we see a clear relationship between case numbers and municipal aggressiveness.^13^

### Ideology

**Figure 7.**
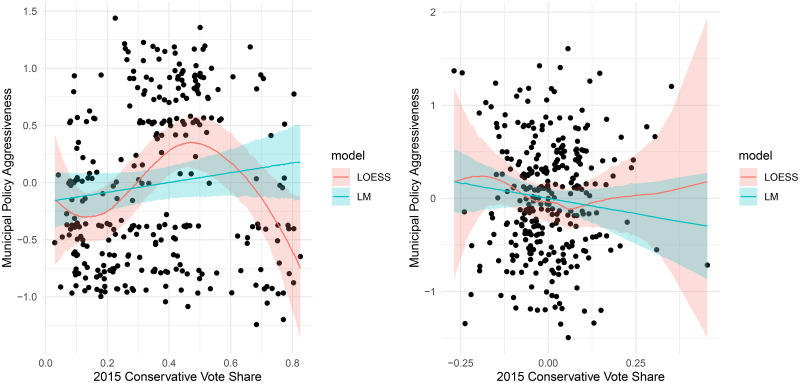
Municipal policy aggressiveness by local ideology.

Finally, Figure 7 plots the relationship between policy aggressiveness and municipal ideological complexion, which we measure using an estimate of Conservative Party vote share in the 2015 federal election. The model was estimated with a second-degree local polynomial regression (LOESS) model.^14^ Perhaps surprisingly, municipalities with middling levels of conservative vote share have the most aggressive policy stances, on average. Conservative vote share explains about 10 percent of the variation in aggressiveness of COVID-19 response, reliably more than the linear model (R^2^= 0.01). This nonlinear relationship does not hold up when controlling for province, however, as shown in the right-hand panel of Figure 7.

## Conclusion

Understanding the factors that shape variation in municipal public policy is a vibrant area of current research (Einstein and Kogan, 2016). In this note, we described municipal COVID-19 responses and found that municipal population size and local COVID-19 case totals are strongly related to municipal policy aggressiveness. In general, however, Canadian municipalities have responded to the pandemic in remarkably consistent ways.

We also found modest variation in policy responses by province and ideology. These differences reflect features of Canadian political geography that are not yet well understood, especially the role of regional clustering in local policy diffusion. We thus see an important role for COVID-19 research as part of a larger agenda on the determinants of Canadian municipal public policy.

## Notes

1 See the Appendix (available online) for questions, breakdowns, and other details.

2 See https://citywatchcanada.ca/.

3 For technical details about how the model was estimated and an extended discussion about model identification and more about convergence, see the online Appendix.

4 Note that the city hall indicator has no credible interval around it because we set its value deterministically for the purposes of identification. For more on this, see the online Appendix.

5 Just with any scaling procedure, the latent variable has no inherent meaning. We treat it as policy aggressiveness because that interpretation is consistent with the results of the model. Again, as with any scaling model, unless we introduce other external measures, there is no way to definitively determine the true “meaning” of the latent variable.

6 NWT is not included in this plot because we did not receive any responses from Yellowknife, the only municipality in NWT above 9,000 population.

7 We used Monte Carlo integration to develop these estimates. For more on this approach, see the online Appendix.

8 See the online Appendix for detail on this follow-up research.

9 Ontario's estimated latent aggressiveness is reliably higher than Alberta, BC, Manitoba, New Brunswick, Newfoundland and Labrador, Nova Scotia and Quebec. In these cases, the posterior probability that Ontario's estimate is greater than the other province is greater than .95.

10 Given the highly skewed distribution of municipal population sizes, we use logged population size in the figure and analysis.

11 One review suggested that the results appeared to be nonlinear and/or driven by a few outliers. To our eyes, the seven high leverage points with *log*(pop) > 13.25 will be mostly offset by the six points below the line where 12.5 < *log*(pop) < 13.25. The posterior probability that the adjusted *R*^2^ of a LOESS model is bigger than the adjusted *R*^2^ for the linear model is 0.738 for all of the data, 0.984 for the data where *log*(pop) < 13.25 and 0.7 for the data where *log*(pop) < 12.5. The statistically reliable result for the subset where *log*(pop) < 13.25 goes away when we remove the offsetting over-predicted points 12.5 < *log*(pop) < 13.25. Thus, we argue that there is little evidence of true nonlinearity in this result. The linear model coefficient for the full data is 0.149 with posterior probability greater than zero of 0.995. The linear model coefficient for the *log*(pop) < 12.5 subset is 0.172 with posterior probability greater than zero of 0.991. This would suggest the linear finding is quite robust.

12 The relationships are statistically unreliable in both cases, *b* = { 0.029, 0.027} with 75.7 percent, 84.2 per cent of the posterior distribution of the coefficients above zero.

13 The relationship is statistically reliable: *b* = 0.03 with 1.0 per cent of the posterior distribution to the left of zero.

14 We controlled for province by residualizing the latent mean and conservative vote based on province. For more on this see the online Appendix.

## Supplementary material

For supplementary material accompanying this paper visit https://doi.org/10.1017/S000842392000044X. click here to view supplementary material
