# Supplementary material for: Measuring and Comparing Municipal Policy Responses to COVID-19
Source: Can J Polit Sci. 2020 May 8:1–12. doi: 10.1017/S000842392000044X (PMC7251256; doi:10.1017/S000842392000044X)
Supplement: Supplementary file 1 [file S000842392000044Xsup001.docx]

# Measuring and Comparing Municipal Policy Responses to COVID-19

# Online Appendix

## Canadian Municipal Barometer Survey

The Canadian Municipal Barometer COVID-19 survey was fielded between April 3 and April 17, 2020. Invitations were sent to all of the mayors and councillors for whom the CMB research team was able to collect email addresses - more than 95% of the total population of mayors and councillors in municipalities above 9,000 population in Canada. Of the 3,419 email invitations received by municipal leaders, a total of 551 completed the survey, for an overall response rate of 16 percent.

For additional insight on some of the findings in the CMB survey, we conducted electronic correspondence with four experts after the survey closed - one current municipal mayor, one current municipal councillor, and two individuals with extensive experience as municipal and/or provincial public servants. To better understand the “emergency management” variable, we also selected a random sample of twenty municipalities, ten in Ontario and ten outside Ontario, and read public press releases and media coverage of the “state of emergency” declarations in each of the twenty municipalities. The twenty municipalities we selected were: Newmarket, Leamington, Centre Wellington, Burlington, Mississippi Mills, Toronto, Richmond Hill, Strathroy-Caradoc, Elliot Lake, Wellesley, Red Deer, Kamloops, Lévis, Montreal, Delta, Sherbrooke, Camrose, Pincourt, Leduc, and North Saanich.

Below, we provide complete question texts and response breakdowns for each of the questions used in this paper. Note that complete datasets are publicly available at the CMB dataverse: (<https://doi.org/10.5683/SP2/KIX0MD>).

#### emergency

“Has your municipality declared a state of emergency?”

|  | Freq | % |
| --- | --- | --- |
| **No (0)** | 265 | 50.19 |
| **Yes (1)** | 258 | 48.86 |
| **Don’t know (9)** | 5 | 0.95 |
| **Total** | 528 | 100.00 |

#### cases

“To the best of your knowledge, how many reported COVID-19 cases have occurred so far in your municipality?”

|  | Freq | % |
| --- | --- | --- |
| **0 (1)** | 81 | 15.34 |
| **1-9 (2)** | 172 | 32.58 |
| **10-24 (3)** | 60 | 11.36 |
| **25-49 (4)** | 46 | 8.71 |
| **50-99 (5)** | 27 | 5.11 |
| **100-249 (6)** | 41 | 7.77 |
| **250-499 (7)** | 16 | 3.03 |
| **More than 500 (8)** | 17 | 3.22 |
| **Don’t know (9)** | 68 | 12.88 |
| **Total** | 528 | 100.00 |

#### aggressiveness

"Some municipalities have chosen to respond to COVID-19 with very aggressive social distancing policies, facility and event closures, and other measures. Others currently have less aggressive restrictions in place.

How would you describe your municipality’s current approach to the COVID-19 pandemic?"

|  | Freq | % |
| --- | --- | --- |
| **Not at all aggressive (1)** | 15 | 2.85 |
| **Moderately aggressive (2)** | 288 | 54.75 |
| **Extremely aggressive (3)** | 222 | 42.21 |
| **Don’t know (9)** | 1 | 0.19 |
| **Total** | 526 | 100.00 |

#### status_cityhall

"What is the status of the following facilities and services in your municipality?

City Hall (or equivalent facility)"

|  | Freq | % |
| --- | --- | --- |
| **Fully closed (1)** | 270 | 51.43 |
| **Partially closed or reduced service (2)** | 252 | 48.00 |
| **Open / service as usual (3)** | 3 | 0.57 |
| **Not applicable to my municipality (4)** | 0 | 0.00 |
| **Don’t know (9)** | 0 | 0.00 |
| **Total** | 525 | 100.00 |

#### status_recreation

"What is the status of the following facilities and services in your municipality?

Recreation facilities"

|  | Freq | % |
| --- | --- | --- |
| **Fully closed (1)** | 515 | 98.10 |
| **Partially closed or reduced service (2)** | 8 | 1.52 |
| **Open / service as usual (3)** | 0 | 0.00 |
| **Not applicable to my municipality (4)** | 2 | 0.38 |
| **Don’t know (9)** | 0 | 0.00 |
| **Total** | 525 | 100.00 |

#### status_parks

"What is the status of the following facilities and services in your municipality?

Parks and playgrounds"

|  | Freq | % |
| --- | --- | --- |
| **Fully closed (1)** | 365 | 69.52 |
| **Partially closed or reduced service (2)** | 154 | 29.33 |
| **Open / service as usual (3)** | 5 | 0.95 |
| **Not applicable to my municipality (4)** | 1 | 0.19 |
| **Don’t know (9)** | 0 | 0.00 |
| **Total** | 525 | 100.00 |

#### status_libraries

"What is the status of the following facilities and services in your municipality?

Public libraries"

|  | Freq | % |
| --- | --- | --- |
| **Fully closed (1)** | 490 | 93.33 |
| **Partially closed or reduced service (2)** | 21 | 4.00 |
| **Open / service as usual (3)** | 0 | 0.00 |
| **Not applicable to my municipality (4)** | 10 | 1.90 |
| **Don’t know (9)** | 4 | 0.76 |
| **Total** | 525 | 100.00 |

#### status_transit

"What is the status of the following facilities and services in your municipality?

Public transit"

|  | Freq | % |
| --- | --- | --- |
| **Fully closed (1)** | 43 | 8.19 |
| **Partially closed or reduced service (2)** | 279 | 53.14 |
| **Open / service as usual (3)** | 75 | 14.29 |
| **Not applicable to my municipality (4)** | 119 | 22.67 |
| **Don’t know (9)** | 9 | 1.71 |
| **Total** | 525 | 100.00 |

#### gov_council

"What is the status of the following governance processes in your municipality?

Council meetings"

|  | Freq | % |
| --- | --- | --- |
| **Cancelled (1)** | 16 | 3.05 |
| **In camera (2)** | 30 | 5.71 |
| **Switched to virtual / teleconference (3)** | 465 | 88.57 |
| **Proceeding as usual (4)** | 12 | 2.29 |
| **Not applicable to my municipality (8)** | 0 | 0.00 |
| **Don’t know (9)** | 2 | 0.38 |
| **Total** | 525 | 100.00 |

#### gov_committee

"What is the status of the following governance processes in your municipality?

Committee meetings"

|  | Freq | % |
| --- | --- | --- |
| **Cancelled (1)** | 205 | 39.05 |
| **In camera (2)** | 17 | 3.24 |
| **Switched to virtual / teleconference (3)** | 298 | 56.76 |
| **Proceeding as usual (4)** | 2 | 0.38 |
| **Not applicable to my municipality (8)** | 1 | 0.19 |
| **Don’t know (9)** | 2 | 0.38 |
| **Total** | 525 | 100.00 |

#### gender

A total of 34 percent of the men and women who completed the CMB COVID-19 survey were women, compared with 33% in the population.

|  | Freq | % |
| --- | --- | --- |
| **Man** | 314 | 60.27 |
| **Woman** | 157 | 30.13 |
| **Other response** | 38 | 7.29 |
| **Refused** | 12 | 2.30 |
| **Total** | 521 | 100.00 |

#### province

The distribution of respondents in the CMB survey matches the overall distribution of municipal mayors and councillors in the population very well, with no more than 2% differences between population and respondents in all categories except Quebec, where respondents’ proportion in the survey is 3.5% lower than their proportion of the larger population.

|  | Freq | % |
| --- | --- | --- |
| **AB** | 67 | 11.59 |
| **BC** | 64 | 11.07 |
| **MB** | 22 | 3.81 |
| **NB** | 17 | 2.94 |
| **NFLD** | 12 | 2.08 |
| **NS** | 26 | 4.50 |
| **ON** | 215 | 37.20 |
| **PEI** | 2 | 0.35 |
| **QC** | 142 | 24.57 |
| **SK** | 10 | 1.73 |
| **YT** | 1 | 0.17 |
| **Total** | 578 | 100.00 |

#### population size

Respondent distributions in our sample across population size are very close to respondent distributions in the population of municipal mayors and councillors.

|  | Freq | % |
| --- | --- | --- |
| **9,000-14999** | 185 | 32.01 |
| **15000-24999** | 124 | 21.45 |
| **25000-49999** | 93 | 16.09 |
| **50000-99999** | 56 | 9.69 |
| **100000-499999** | 79 | 13.67 |
| **500000+** | 41 | 7.09 |
| **Total** | 578 | 100.00 |

## Additional Plots: Variation in Policy Choices

Here we consider variation across municipal population size, province, health unit and number of cases. Figure 8 shows the proportion of fully closed facilities (or emergency status declared) by province. The provinces are ordered by their average across all of the indicators; on average, Ontario appears to have the most aggressive response. We will return to this later with a more sophisticated modeling strategy. There does appear to be some structure in the data here - provinces closer to the top have municipalities which have, on average, closed more services than those toward the bottom.

Figure 9 shows policy implementation by city population category. The patterns here are interesting and provide context to the different ways in which policy adoption adapts to the particular municipal context. Here, parks and city hall closures and emergency status are slightly more likely to be invoked in municipalities with greater populations, but transit is more likely to be closed in municipalities with smaller populations - likely due to the necessity of having some public transit available to enable frontline workers to travel to their jobs in large metropolitan areas.


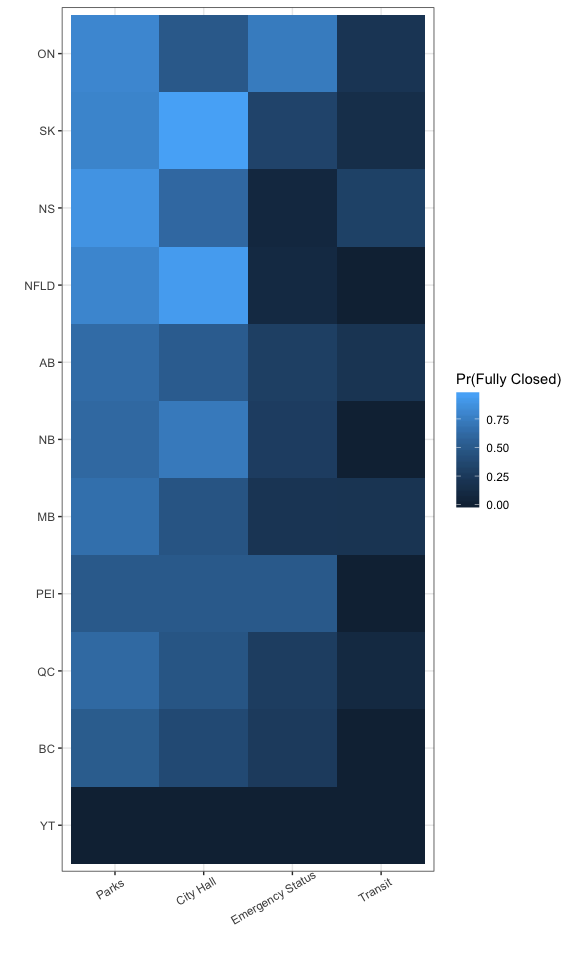


Figure 8: COVID-19 Response by Province


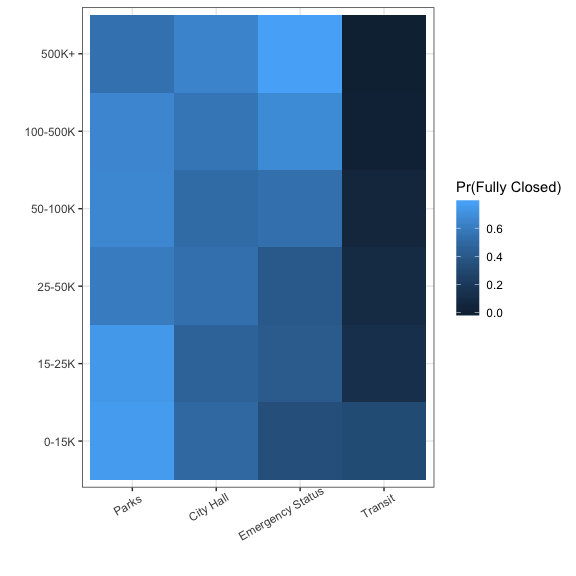


Figure 9: COVID-19 Response by Population


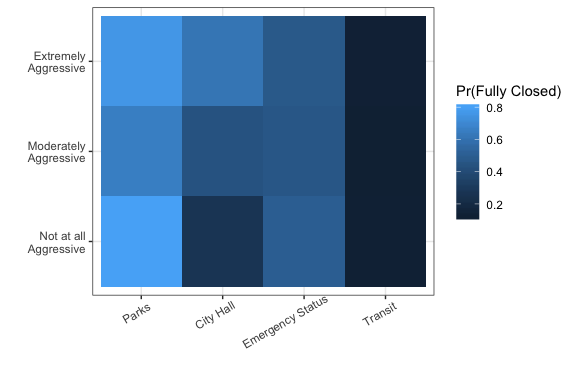


Figure 10: COVID-19 Response by Perceived Aggressiveness of Response

# Technical Discussion of Model

## Identification

There are three common ways of identifying the statistical model. Identification is the process whereby we constrain the model just enough to ensure that there are not multiple equally good solutions. In our OLS models, the identifying restrictions that we put in place are that there are at least as many observations as there are variables in the model and that there is no perfect collinearity. In IRT models, more needs to be done to ensure that other solutions do not arise.

First, for any solution we come up with, there would be an equally good mirror-image solution. For example, if we estimated the discrimination parameter to be 2 and an ideal point to be 1 with a difficulty parameter of 0, then we could get exactly the same value by multiplying both the coefficients and ideal points by -1.

Second, there is no information in the data about the scale (i.e., variance) of the latent variable. Just like above, since the coefficients and the latent variable are both unobserved, changes in the scale of one estimate can be compensated for by changes in the scale of the other. Both of these are issues that we have to solve to make sure that the markov chain is exploring a single posterior distribution.

There are three common ways to identify measurement models of this kind. The way we choose to do it is by setting a single coefficient to a fixed value. Any fixed value would work, but 1 is a sort of reasonable default. This actually solves both problems - fixing the scale of the coefficients and thus the latent variable. It also forces a direction on the coefficients and thus the latent variable, too - it prevents the markov chain from exploring a mirror image posterior distribution. Another common means for identification is to fix the scale of the latent variable in the model. For each iteration of the markov chain, the latent variable would be standardized to have mean zero and unit variance. We also have to set an additional sign constraint on at least one of the coefficients to prevent exploration of a mirror image solution. Another option is to only use sign constraints and then to post-process the chains after the fact to standardize the latent variable and adjust the model parameters accordingly. We choose the first way for simplicity and computational efficiency.

## Priors

The priors in the model may seem somewhat informative, but they are relatively conventional priors for these kinds of models. The latent variable has a prior of $\mathcal{N}(0,1)$. Since one of the reliable items has a coefficient set to 1 for identification purposes, we would expect the ideal points to vary probably between $F^{-1}(.05)=$ -2.94 and $F^{-1}(.95)=$ 2.94where $F(\cdot)$ is the CDF of the logistic distribution. These values are not particularly unusual values for a $\mathcal{N}(0,1)$ distribution. The coefficient priors are $\mathcal{N}_{2}(\mu,\Sigma)$ with $\mu=\{0,0\}$ and $\Sigma=\left[ \begin{matrix} 10 & 0 \\ 0 & 10 \end{matrix} \right]$. Given the scale of the other coefficient and the presumed scale of the latent variable, then we would expect the coefficients to be well within the range of a distribution with these characteristics.

## Estimation

The model we discussed in the article was estimated with JAGS v4.3.0 (Plummer 2017) through R and the rjags package (Plummer 2018). The code for the full and null models is below:

status.mod <- "model{
 for(i in 1:n){
 chstat[i] ~ dbern(p[munid[i], 1])
 parkstat[i] ~ dbern(p[munid[i], 2])
 emergency[i] ~ dbern(p[munid[i], 3])
 transtat[i] ~ dcat(p3[munid[i], 1:3])
 }
 for(i in 1:nm){
 logit(p[i,1]) <- b[1,1] + b[1,2]*latent[i]
 logit(p[i,2]) <- b[2,1] + b[2,2]*latent[i]
 logit(p[i,3]) <- b[4,1] + b[4,2]*latent[i]
 for(j in 1:2){
 logit(q.trans[i,j]) <- kappa3[j] - b[5,2]*latent[i]
 }
 p3[i,1] <- q.trans[i,1]
 p3[i,2] <- q.trans[i,2] - q.trans[i,1]
 p3[i,3] <- 1-q.trans[i,2]
 latent[i] ~ dnorm(0,1)
 }
 for(j in 2:5){
 b[j, 1:2] ~ dmnorm(b0[1:2], B0[1:2,1:2])
 }
 k[1:2] ~ dmnorm(b0[1:2], B0[1:2,1:2])
 kappa3[1:2] <- sort(k[1:2])
}
"

null.mod <- "model{
 for(i in 1:n){
 chstat[i] ~ dbern(p[munid[i], 1])
 parkstat[i] ~ dbern(p[munid[i], 2])
 emergency[i] ~ dbern(p[munid[i], 3])
 transtat[i] ~ dcat(p3[munid[i], 1:3])
 }
 for(i in 1:nm){
 logit(p[i,1]) <- b[1,1]
 logit(p[i,2]) <- b[2,1]
 logit(p[i,3]) <- b[4,1]
 for(j in 1:2){
 logit(q.trans[i,j]) <- kappa3[j]
 }
 p3[i,1] <- q.trans[i,1]
 p3[i,2] <- q.trans[i,2] - q.trans[i,1]
 p3[i,3] <- 1-q.trans[i,2]
 latent[i] ~ dnorm(0,1)
 }
 for(j in 2:5){
 b[j, 1:2] ~ dmnorm(b0[1:2], B0[1:2,1:2])
 }
 k[1:2] ~ dmnorm(b0[1:2], B0[1:2,1:2])
 kappa3[1:2] <- sort(k[1:2])
}
"

In these models, munid is a municipal id counter that goes from 1-306, identifying each municipality. Even though there are multiple respondents in each municiaplity, we are estimating a single ideal point for each municipality.

## Convergence and Model Comparison

We tune the adaptive rejection sampler for 2500 iterations, then burn-in the model for 10,000 iterations and sample 2,500 iterations from each of two chains that we summarise below. The model appears to have converged. The multivariate PSRF for the full model is 1.14 and the upper bound of the biggest individual PSRF was only approximately 1.1 with 318 model parameters. The Geweke diagnostic also shows generally good results with the $z$-statistics generally falling within the confidence bounds of the standard normal distribution.

The model also seems to provide some predictive power. In addition to the model described above, we also estimated a null model - one with only intercept(s) for the indicators. Comparing the deviance information criterion (DIC) values for the null and full model gives us a sense of the value added that the latent variable provides. The DIC for the null model is 2741.235 and for the full model it is 2555.081. As with other information criterion comparisons, smaller values are better. The difference in the DIC values of 186.154 favors the full model indicating that a unidimensional structure model fits better than one that assumes no underlying structure.

# Predictive Models with Monte Carlo Integration.

In the results above, we employ two different kinds of models - an Item Response Theory model to identify the dependent variable and a set of bivariate predictive models to help understand the correlates of policy responsiveness. One way of handling this would be to simply build a full Bayesian structural equation model. This would allow us to incorporate both kinds of models into one. However, in this case, we would need several different SEMs to deal with the different predictors mentioned in the article. In each of these, the incorporation of predictors of the latent trait would change the latent traits values, even if only slightly. Instead of doing this, we opt for the Monte Carlo integration approach.

The Monte Carlo integration approach takes the following steps.

1. Take the first iteration of the Markov Chain for the latent variable and call these $\text{latent}^{(1)}$.
2. Estimate the following model: $\text{latent}^{(1)}=b_{0}+b_{1}x+e$.
3. Take a single draw from the distribution $\mathcal{N}_{2}(B,\hat{V}(B))$, where $B=\{b_{0},b_{1}\}$ and $\hat{V}(B)$ is the variance-covariance matrix of the estimators. We store this single draw from the bivariate normal distribution as the first row of $\beta$. 4. Repeat by replacing with $\text{latent}^{(1)}$ with $\text{latent}^{(t)}$ for $t=1,\ldots,\#\text{Iterations}$.

At the end of this process, we withh have a $\#Iterations\times2$ posterior distribution of coefficients from the model above. We can summarise that distribution, particularly looking for the posterior probability of being above or below zero. This posterior probability amounts to something like a Bayesian $p$-value. If a large proportion of the posterior distribution is above (or below) zero, say 95%, then we could say that the result is statistically reliable. Note that we don’t say significant here becaus significance carries with it frequentist baggage - it particularly identifies a result relative to a null hypothesis in the frequentist inferential paradigm.

All of the models we estimate are simple OLS models, except for the one with conservative vote choice. Here, we estimate a second-degree local polynomial regression model with span = .75; we use the loess() function in R to accomplish this. The inferences we make are based on predictions from this model. We follow the same Monte Carlo integration method described above, but instead of coefficients, we do it with predictions. Here, we draw each prediction from its own univariate normal distribution. To evaluate differences in the linear and non-linear models, we look at the $R^{2}$. We calculate $R^{2}$ for each iteration of the Monte Carlo simulation and then compare the posterior distribution of $R^{2}$ values across the two models.

While this model is generally a bivariate model, we can control for another variable by residualization. In particular, we first estimate these two models:

$$\begin{matrix} \text{Latent} & =\theta\text{Province Dummies}+e^{(\text{latent})} \\ \text{Conservative Vote} & =\theta^{*}\text{Province Dummies}+e^{(\text{cons vote})} \end{matrix}$$

where $\theta$ and $\theta^{*}$ are both vectors of coefficients attached to the provincial dummy variables. Then, we estimate a loess regression of $e^{(\text{latent})}$ on $e^{(\text{cons vote})}$.

# Perceived and Measured Aggressiveness

We asked CMB respondents to describe the aggressiveness of their municipality’s response to COVID-19. Interestingly, as the boxplot in figure 11 shows, these perceptions are almost completely unrelated to our measure of municipal aggressiveness.


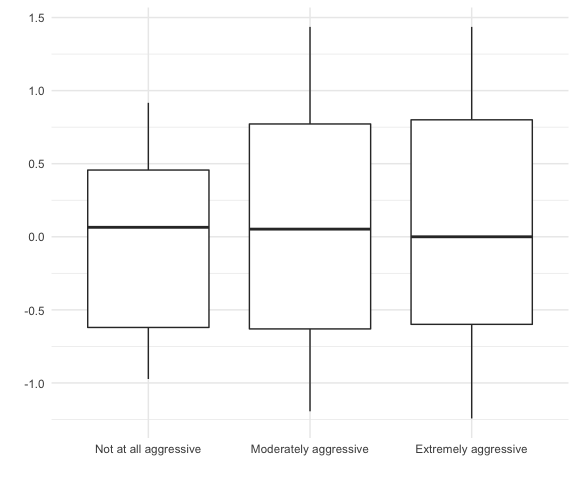


Figure 11: Municipal Policy Aggressiveness by COVID-19 Cases

Why do we see this absence of any relationship between measured and perceived aggressiveness? We cannot fully explore the answer here, but the answer does not appear to be that we are missing some essential indicator from our measure, since all existing evidence suggests that other indicators, such as social distancing enforcement measures, are strongly correlated with the indicators we use here.^[[1]](#footnote-1)^ Nor do we see clear patterns of perceived aggressiveness across provinces, case totals, or municipal population size. Perceived aggressiveness might therefore be more closely related to a respondent’s local environment and comparisons with neighbouring municipalities or regions.

1. <https://citywatchcanada.ca/> [↑](#footnote-ref-1)
